# Confirmation of the pathogenicity of a mutation p.G337C in the *COL1A2* gene associated with osteogenesis imperfecta: Erratum

**DOI:** 10.1097/MD.0000000000008442

**Published:** 2017-10-20

In the article, “Confirmation of the pathogenicity of a mutation p.G337C in the *COL1A2* gene associated with osteogenesis imperfecta”,^[1]^ which appeared in Volume 96, Issue 39 of *Medicine*, the affiliations are out of order. They should appear as:

^a^Department of Pain Management, Shandong Provincial Hospital Affiliated to Shandong University, Jinan, ^b^Department of Pediatrics, Shandong Provincial Hospital Affiliated to Shandong University, Jinan, ^c^Linyi County Traditional Chinese Medicine Hospital, Dezhou, ^d^Department of Endocrinology and Metabolism, Shandong Provincial Hospital Affiliated to Shandong University, Jinan, ^e^Institute of Endocrinology, Shandong Academy of Clinical Medicine, Jinan, ^f^Shandong Clinical Medical Center of Endocrinology and Metabolism, Jinan, Shandong, China.
